# Developing and testing a Corona VaccinE tRiAL pLatform (COVERALL) to study Covid-19 vaccine response in immunocompromised patients

**DOI:** 10.1186/s12879-022-07621-x

**Published:** 2022-07-28

**Authors:** Katharina Kusejko, Frédérique Chammartin, Daniel Smith, Marc Odermatt, Julian Schuhmacher, Michael Koller, Huldrych F. Günthard, Matthias Briel, Heiner C. Bucher, Benjamin Speich, the Swiss HIV Cohort Study, I. Abela, K. Aebi-Popp, A. Anagnostopoulos, M. Battegay, E. Bernasconi, D. L. Braun, H. C. Bucher, A. Calmy, M. Cavassini, A. Ciuffi, G. Dollenmaier, M. Egger, L. Elzi, J. Fehr, J. Fellay, H. Furrer, C. A. Fux, H. F. Günthard, A. Hachfeld, D. Haerry, B. Hasse, H. H. Hirsch, M. Hoffmann, I. Hösli, M. Huber, C. R. Kahlert, L. Kaiser, O. Keiser, T. Klimkait, R. D. Kouyos, H. Kovari, K. Kusejko, G. Martinetti, B Martinez de Tejada, C. Marzolini, K. J. Metzner, N. Müller, J. Nemeth, D. Nicca, P. Paioni, G. Pantaleo, M. Perreau, A. Rauch, P. Schmid, R. Speck, M. Stöckle, P. Tarr, A. Trkola, G. Wandeler, S. Yerly, Swiss Transplant Cohort Study, Patrizia Amico, John-David Aubert, Vanessa Banz, Sonja Beckmann, Guido Beldi, Christoph Berger, Ekaterine Berishvili, Annalisa Berzigotti, Isabelle Binet, Pierre-Yves Bochud, Sanda Branca, Heiner Bucher, Emmanuelle Catana, Anne Cairoli, Yves Chalandon, Sabina De Geest, Olivier De Rougemont, Sophie De Seigneux, Michael Dickenmann, Joëlle Lynn Dreifuss, Michel Duchosal, Thomas Fehr, Sylvie Ferrari-Lacraz, Christian Garzoni, Déla Golshayan, Nicolas Goossens, Fadi Haidar Jörg Halter, Dominik Heim, Christoph Hess, Sven Hillinger, Hans H. Hirsch, Patricia Hirt, Günther Hofbauer, Uyen Huynh-Do, Franz Immer, Michael Koller, Mirjam Laager, Bettina Laesser, Frédéric Lamoth, Roger Lehmann, Alexander Leichtle, Oriol Manuel, Hans-Peter Marti, Michele Martinelli, Valérie McLin, Katell Mellac, Aurélia Merçay, Karin Mettler, Antonia Müller, Nicolas J. Mueller, Ulrike Müller-Arndt, Beat Müllhaupt, Mirjam Nägeli, Graziano Oldani, Manuel Pascual, Jakob Passweg, Rosemarie Pazeller, Klara Posfay-Barbe, Juliane Rick, Anne Rosselet, Simona Rossi, Silvia Rothlin, Frank Ruschitzka, Thomas Schachtner, Urs Schanz, Stefan Schaub, Alexandra Scherrer, Aurelia Schnyder, Macé Schuurmans, Simon Schwab, Thierry Sengstag, Federico Simonetta, Susanne Stampf, Jürg Steiger, Guido Stirnimann, Ueli Stürzinger, Christian Van Delden, Jean-Pierre Venetz, Jean Villard, Julien Vionnet, Madeleine Wick, Markus Wilhelm, Patrick Yerly

**Affiliations:** 1 Institute of Medical Virology, University of Zurich, Zurich, Switzerland; 2 Division of Infectious Diseases and Hospital Epidemiology, University Hospital Zurich, Zurich, Switzerland; 3 Basel Institute for Clinical Epidemiology and Biostatistics, Department of Clinical Research, University Hospital Basel, University of Basel, Basel, Switzerland; 4 Department of Health Research Methods, Evidence, and Impact, McMaster University, Hamilton, Canada; 5 Centre for Statistics in Medicine, Nuffield Department of Orthopaedics, Rheumatology and Musculoskeletal Sciences, University of Oxford, Oxford, UK

**Keywords:** Trial platform, SARS-CoV-2, Immunocompromised, HIV, Transplant patients, REDCap

## Abstract

**Background:**

The rapid course of the severe acute respiratory syndrome coronavirus 2 (SARS-CoV-2) pandemic calls for fast implementation of clinical trials to assess the effects of new treatment and prophylactic interventions. Building trial platforms embedded in existing data infrastructures is an ideal way to address such questions within well-defined subpopulations.

**Methods:**

We developed a trial platform building on the infrastructure of two established national cohort studies: the Swiss human immunodeficiency virus (HIV) Cohort Study (SHCS) and Swiss Transplant Cohort Study (STCS). In a pilot trial, termed Corona VaccinE tRiAL pLatform (COVERALL), we assessed the vaccine efficacy of the first two licensed SARS-CoV-2 vaccines in Switzerland and the functionality of the trial platform.

**Results:**

Using Research Electronic Data Capture (REDCap), we developed a trial platform integrating the infrastructure of the SHCS and STCS. An algorithm identifying eligible patients, as well as baseline data transfer ensured a fast inclusion procedure for eligible patients. We implemented convenient re-directions between the different data entry systems to ensure intuitive data entry for the participating study personnel. The trial platform, including a randomization algorithm ensuring balance among different subgroups, was continuously adapted to changing guidelines concerning vaccination policies. We were able to randomize and vaccinate the first trial participant the same day we received ethics approval. Time to enroll and randomize our target sample size of 380 patients was 22 days.

**Conclusion:**

Taking the best of each system, we were able to flag eligible patients, transfer patient information automatically, randomize and enroll the patients in an easy workflow, decreasing the administrative burden usually associated with a trial of this size.

## Background and purpose

In 2020, the coronavirus disease 2019 (Covid-19) pandemic evoked unprecedented challenges concerning nearly all parts of health care systems worldwide [1]. With almost daily updated information concerning epidemiological and immunological knowledge about the virus, authorities continue to need up-to-date evidence for decision making in regard to the Covid-19 pandemic. Carefully designed and conducted clinical trials are key to understand different aspects of the on-going epidemic, including testing, treating and vaccination strategies in different subpopulations [2]. Given the rapid course of the events, clinical researchers and trial support units worldwide were challenged to set up trials very fast, including the corresponding data collection tools [3]. While usually the process of setting up a trial starts with a clear study plan, then followed by the implementation of the data infrastructure, the process needed to be more flexible in the case of Covid-19 in order to act fast.

Immunocompromised patients, such as transplanted patients or people living with human immunodeficiency virus (HIV), were mostly left out in the first trials investigating immune response to vaccines against severe acute respiratory syndrome coronavirus 2 (SARS-CoV-2) [4, 5]. Thus, this group of patients form a potentially vulnerable group with many open questions concerning the efficacy and safety of SARS-CoV-2 vaccines [6–8]. At the time of our study design, only few studies were published looking specifically into transplanted patients or people living with HIV: In the case of people receiving a solid organ transplant, Boyarsky et al. reported SARS-CoV-2 antibody responses of 658 patients that received a solid organ transplant in the US [6, 9]. In this study, solid organ transplant patients that already received a SARS-CoV-2 vaccine were recruited through social media to participate in a prospective cohort to measure the antibody response. In the case of people living with HIV, Woldemeskel et al. studied cellular and humoral immune response in 12 people living with HIV and 17 HIV-negative controls, they found no significant difference in the titers of SARS-CoV-2 spike antibodies between cases and controls in this study population [10]. Similarly, Ruddy et al. observed high SARS-CoV-2 antibody titers in 14 people living with HIV [11].

Cohort studies form an ideal research framework to perform nested clinical trials, such as studying the efficacy and safety of new treatment or prophylactic options. The main advantage is that in addition to trial specific data, longitudinal information about these participants are available and ready to be used for more in-depth analysis [12, 13]. In addition, contact details of the potential study participants are already available, which accelerates a trial set-up. This was shown by the Rotterdam Study, a prospective cohort study, where more than 8000 study participants were invited to fill out COVID-19 specific questions already in April 2020 [14].

**Fig. 1:**
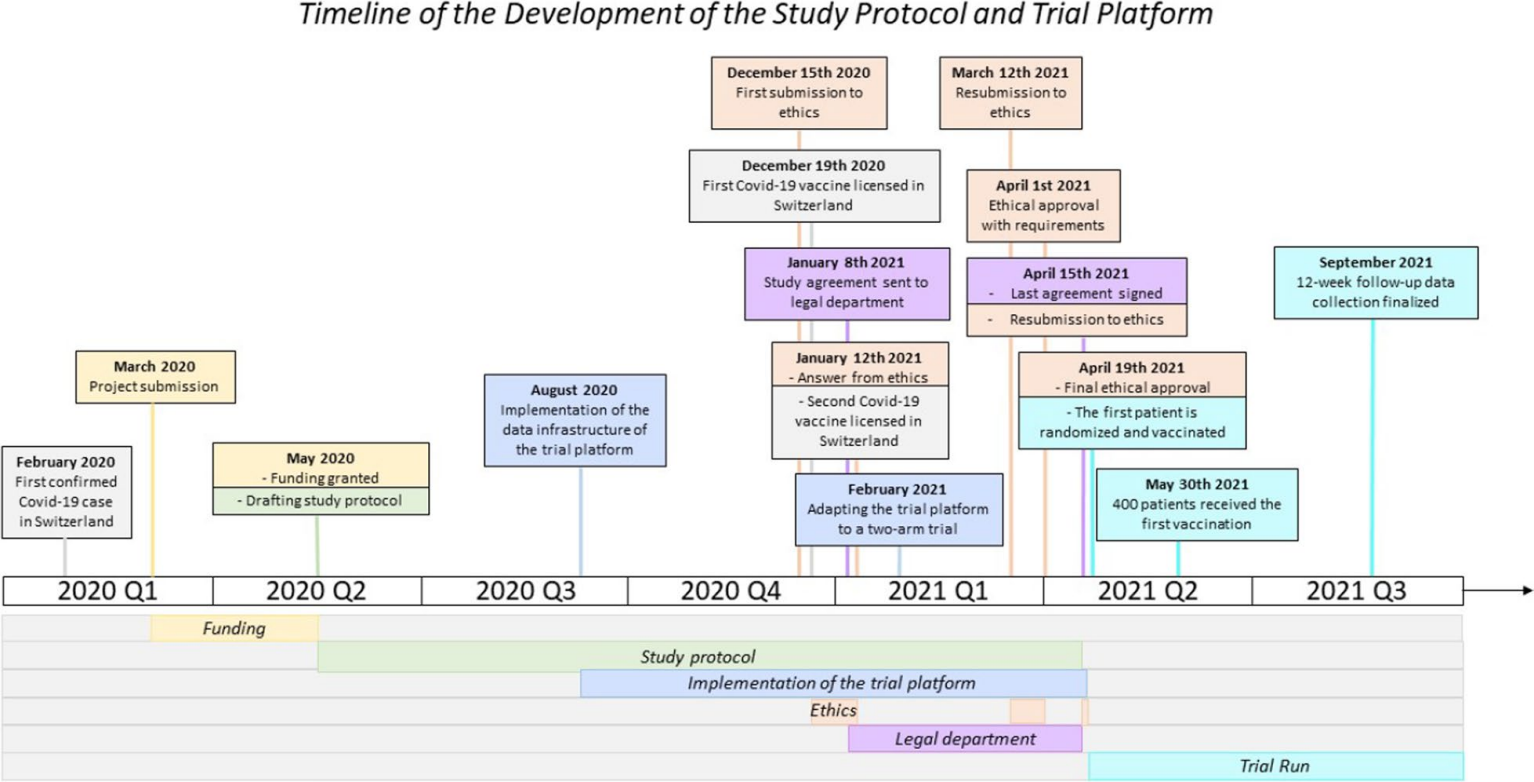
Overview of the timeline for development of the trial platform: From the first confirmed Covid-19 case in Switzerland to the finalization of the 12 week follow-up data collection

In this project, we aimed to develop a trial platform within the framework of the Swiss HIV Cohort Study (SHCS) and Swiss Transplant Cohort Study (STCS), two well-established nation-wide cohort studies [15, 16]. We aimed to develop an interoperable system embedded in the data collection of two running cohort studies ensuring transfer of baseline data. We aimed to include intuitive redirections between the systems to keep the administrative burden of the involved physicians and study nurses to a minimum. In a pilot project, the Corona VaccinE tRial pLatform (COVERALL) was set up to assess the vaccine efficacy of the first two licensed SARS-CoV-2 vaccines in Switzerland and the functionality of the trial platform. Figure 1 summarizes the timeline of this project.

## Methods

### Study design of the Swiss HIV Cohort Study

The Swiss HIV Cohort Study (SHCS) is a prospective multicenter cohort study enrolling adult people living with HIV in Switzerland [15, 17]. The study was launched in 1988, is approved by all local research ethics committees. Written informed consent was obtained from all participants. Clinical, laboratory, epidemiological and lifestyle information is collected during bi-annual follow-up visits in all patients. All medication including HIV treatment, co-medication and vaccinations are reported in the SHCS. Starting in April 2020, the SHCS dynamically introduced and adapted SARS-CoV-2 relevant information, including information regarding SARS-CoV-2 testing (polymerase chain reaction, tests for SARS-CoV-2 antigen, and antibody), infection, as well as vaccination. In 2018, rollout of an electronic data capture tool started, enabling physicians and study nurses to enter patient information electronically into the database. The SHCS data capture tool uses the Django Web Framework, a high-level Python-based web framework built for development of applications, which manages all parts of application development, from database back-end to user-friendly front-end [18]. The Django Framework offers the possibility for the development of customized tools for data entry, data monitoring, benchmarking and quality checks. With its modular design, one can implement independent tools and features within the productive environment and make them visible to specific subsets of users. The SHCS covers around 75% of all individuals on antiretroviral therapy in Switzerland and is representative of the HIV epidemic in this country [15, 17].

### Study design of the Swiss Transplant Cohort Study (STCS)

The Swiss Transplant Cohort Study (STCS) is a multicenter, prospective cohort study, launched in 2008, enrolling all patients receiving a solid organ transplant in Switzerland (transplantation centers: Basel, Bern, Geneva, St. Gallen, Lausanne and Zurich) [16]. The STCS was approved by the local research ethics committees of all participating institutions, and all participants provided their written informed consent. The STCS uses a data capture tool enabling study nurses at the transplant centers to enter patient information electronically. In line and close collaboration with the SHCS, the STCS decided to migrate the legacy system to the Django Web Framework and will soon use it for capturing clinical data [18]. The STCS also implements the requirements of the Swiss Transplantation Act for nationwide follow-up monitoring of all transplanted patients since 2008. For this purpose, a minimal dataset, i.e., baseline information about the transplantation and patient characteristics, is collected from all solid organ transplant recipients in Switzerland according to the legal requirements. For patients who provided their written informed consent to take part in the STCS, the full research dataset, i.e., information about follow-up visits and biological samples, is collected. In regard to Covid-19, the STCS implemented a targeted system to capture all Covid-19 cases in organ transplant patients since the beginning of the pandemic.

### Design of the pilot trial: Corona VaccinE tRiAL pLatform (COVERALL)

We set up the COVERALL trial platform and customized it for the first pilot trial to test the functionality of the trial platform (see Fig. 1 for an overview of the study timeline). The main focus was to determine the duration of the trial set up, i.e., time from deciding which interventions will be tested until the first patient is randomized, as well as the time of patient recruitment, i.e., time from activation of the first study site until (i) 40 patients and (ii) 380 patients (the targeted trial population) were randomized. Moreover, we were interested in the following pre-specified outcomes: Patient consent rate, proportion of missing data at baseline and proportion of missing data for clinical outcomes. The COVERALL pilot trial is a parallel two-arm, open-label, non-inferiority randomised clinical trial with the aim two compare the first two licensed SARS-CoV-2 vaccines in Switzerland [20]. Eligible patients enrolled in the SHCS or STCS were approached by study personnel, and written informed consent for participation in COVERALL was obtained.

### Data infrastructure

**Fig. 2:**
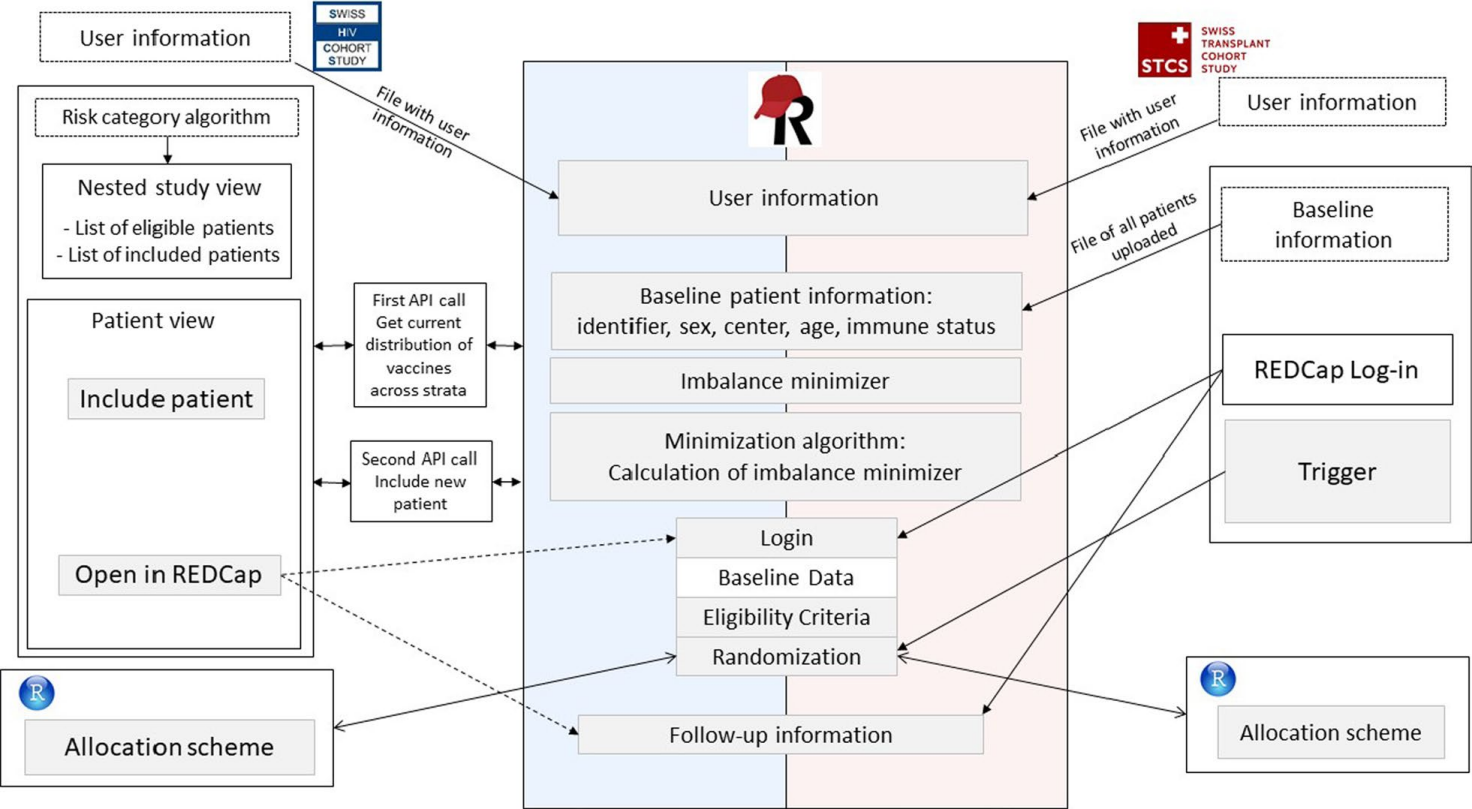
Workflow overview. The basic layout of the infrastructure: Both system, the Swiss HIV Cohort Study (SHCS) and Swiss Transplant Cohort Study (STCS) interact with the newly set-up trial platform on Research Electronic Data Capture (REDCap). Various application programming interfaces (API) and triggers enable communication between these systems

The trial platform uses the data infrastructure of the two well-established systems of the SHCS and STCS. These systems were connected to a new data collection tool developed with Research Electronic Data Capture (REDCap). The platform was customized for a first pilot trial comparing the messenger ribonucleic acid (mRNA) vaccines Comirnaty® and Spikewax® in the SHCS and STCS, termed COVERALL [20]. The innovation of this project is to perform automatic patient selection, randomization, and automatic transfer of baseline data to the trial platform by using the available data infrastructures (SHCS and STCS) and the REDCap data collection tool. We summarized the workflow in Fig. 2. We outline the detailed steps of the data infrastructure in the following paragraphs.

**Fig. 3:**
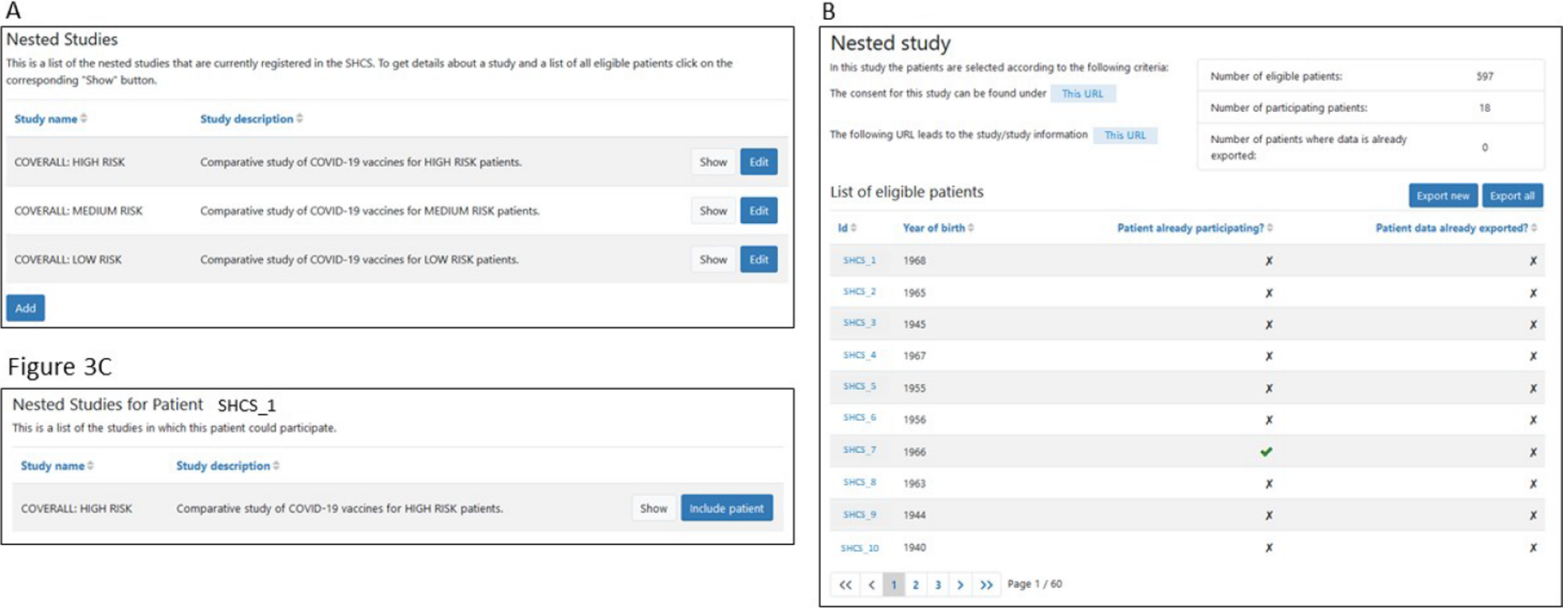
In the electronic data capture system of the Swiss HIV Cohort Study (SHCS), communication tools with the trial platform are embedded. **A** Nested studies including lists of eligible patients can be created. **B** Inclusion of the eligible patients is tracked in the SHCS. **C** On the specific patient page, nested studies for which the patient is eligible are shown

Flagging patients within the SHCS: In autumn 2020, we extended the SHCS electronic data entry tool by a feature allowing for integrating nested studies in the workflow of a regular SHCS follow-up visit. The users are made aware of the existence of substudies within the SHCS, eligibility of patients is indicated. For COVERALL, a nested trial with three levels of prioritization of patients was added (see Fig. 3A). For this, at COVERALL study launch, an algorithm was executed to assign a priority (high, medium, low) to each active patient, based on the most recent laboratory and clinical information and the current recommendations given by the Federal Office of Public Health:

1. *High risk*: All patients aged at least 75, the most recent CD4 count below 200 cells/µL, or the latest HIV viral load measurement being detectable (> 500 copies/ml).
2. *Medium risk*: All patients (not in the high risk category) with a past coronary heart disease, diabetes, hypertension, metabolic syndrome, low glomerular filtration rate, or high body mass index.
3. *Low risk*: All remaining active patients not in the high or medium risk category.

A list of eligible patients in the three categories was produced and integrated into the Django system, with dynamic updates indicating whether the patient is participating (see Fig. 3B). The nested study tool was set to be visible for all physicians and study nurses participating in the COVERALL study, i.e., only to a subset of the participating SHCS study nurses and physicians. On the patient overview page, the user is made aware of the COVERALL study, and the risk category is indicated. Clicking on the respective link, the user can navigate directly to the nested study view, where patients can be included in the study with only one click (see Fig. 3C).

Patient selection and baseline data transfer for the STCS: Before the COVERALL study launch, baseline data needed for the randomization algorithm (patient STCS identifier, sex, birth year, center, type of organ (kidney or lung), immunosuppressive treatments, date of transplantation) was extracted from the STCS database and uploaded to the REDCap platform.

Log-in for users to REDCap: Before the COVERALL study launch, a list of all participating study nurses and physicians was requested by the participating SHCS and STCS centers. A file including names and e-mail addresses was then uploaded to REDCap to automatically create REDCap user accounts. All users received an automatic email by REDCap with log-in information. User roles including access rights were then assigned to the study nurses and physicians.

**Fig. 4:**
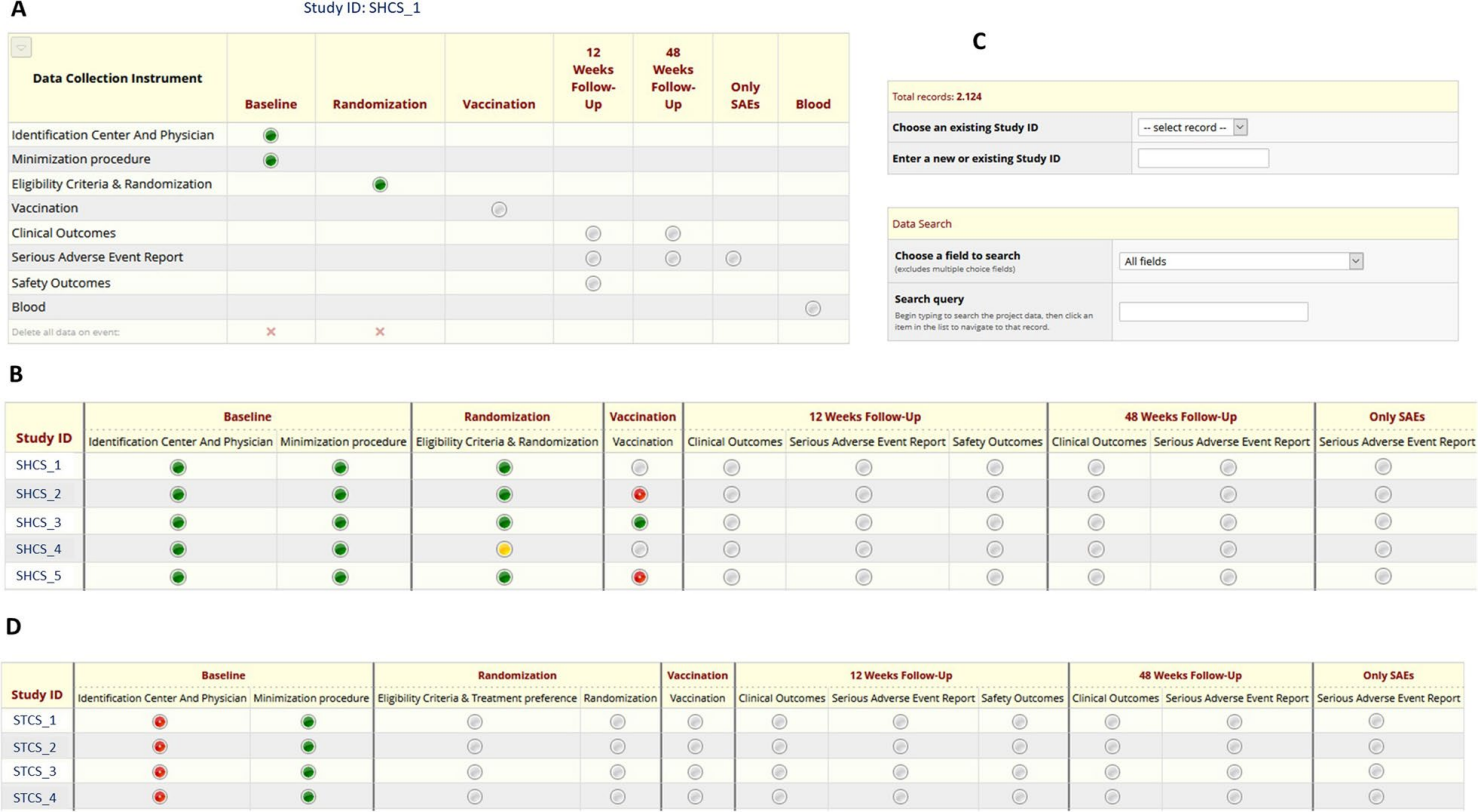
Study overview on the REDCap trial platform: **A** A dashboard for each patient visualizes the progress of the trial. **B** A dashboard specific to the Swiss HIV Cohort Study (SHCS) gives an overview of the study progress, in red highlighting missing items. **C** A search tool implemented in REDCap can be used to navigate to specific data entry forms. **D** A dashboard specific to the Swiss Transplant Cohort Study (STCS) gives overview of the study progress

Inclusion of new SHCS patients and follow-up information: For the SHCS, an application programming interface (API, see details below) was implemented to allow communication between the SHCS electronic data platform (Django) and REDCap. Patients can be added to the COVERALL study in the SHCS Django (see *Flagging patients within the SHCS* above). The user is directly navigated to the REDCap platform and, after log-in, directly redirected to the respective patient (see Fig. 4A). To enter follow-up information, the user can navigate to the patient file using the links in the SHCS Django, or navigate on the REDCap dashboard, where information regarding the progress about inclusion and follow-up process is given (see Fig. 4B). For the STCS, the same API was not yet implemented in STCS Django because the system is not yet operational. Therefore, subject selection and baseline data of all patients was uploaded to REDCap before the COVERALL study launch. To include a new patient or to enter follow-up information, the STCS user needs to directly navigate to the main REDCap interface, log-in, and search for the respective patient STCS identifier in the REDCap search tool (see Fig. 4C). After filling out eligibility and exclusion criteria, the patient was then randomized and was hence included in the COVERALL study. After this, follow-up information and adverse events could be entered. On the study dashboard, progress of the inclusion and follow-up process is continuously monitored (see Fig. 4D).

API and trigger*:* In the SHCS, no patient information was available in REDCap at COVERALL study launch, baseline information is transferred stepwise and automatically only after including the patient via the SHCS Django-REDCap API: For inclusion of a patient, the “Include patient”-button (see Fig. 3C) triggers an API call executing the following tasks:

1. COVERALL study population: All SHCS patient identifiers, including the allocated treatment for already randomized patients, are obtained from REDCap.
2. New patient: Baseline data of the new patient is fetched through the SHCS Django system
3. Balance of treatments: Based on the current study population and the baseline information of the new patient, the treatment, which minimizes the overall imbalance, is calculated, termed “imbalance minimizer” (see *Minimization Algorithm for Randomization* below).
4. Baseline data transfer: Transfer of baseline data of the new patient to REDCap, including the before calculated imbalance minimizer.

After inclusion of patients, the “Include patient”-Button changes to an “Open in REDCap”-Button, providing a direct link to the patient dashboard of the REDCap platform (see Fig. 4A). In the case of the STCS, baseline data of all patients was uploaded before study launch. For every change in an existing record, e.g., verifying inclusion and exclusion criteria for COVERALL, a request to the Django system is triggered, sending a message with the patient SHCS identifier of the modified patient. The patient information is obtained via the API. In case the patient should be randomized, i.e., inclusion criteria and exclusion criteria are correctly filled out, the imbalance minimizer is calculated based on the previously randomized patients. Then the patient can be randomized.

### Minimization algorithm for randomization

The main goal is that patients are randomized to the two available vaccines, Vaccine A (Comirnaty®) or Vaccine B (Spikewax®) such that the imbalance of the two vaccines is minimized regarding the four co-variables:

- Age: ≥ 65 or < 65 years old
- Sex: male or female
- Study center: Zurich, Basel, Bern
- Immunosuppression status:
  - SHCS: CD4 < 200 or CD4 >  = 200 cells/µL
  - STCS: Use of induction immunosuppressive treatment or not

While REDCap offers a built-in tool for randomization, i.e., equal chance for all choices, the minimization algorithm had to be developed outside REDCap. In particular, imbalance minimizer was calculated using the R software [21]: The four co-variables are weighted equally and the treatment leading to the smaller total imbalance in the four categories is chosen [22], i.e., *Treatment A* or *Treatment B*. In case of equal balance, the imbalance minimizer is set to *Both*. The variation (total imbalance) of each category is quantified as sample variance. For the SHCS, the imbalance minimizer is calculated via the API when including the patient via Django (see *API and trigger*). For the STCS, the imbalance minimizer is calculated when the trigger is executed (see *API and trigger*). Based on the imbalance minimizer, the treatment is assigned randomly with unequal probabilities using the sample function in R. In case the imbalance minimizer is ‘Treatment A’, chances to get assigned to ‘Treatment A’ are 80% and chances to get assigned to ‘Treatment B’ are 20%, vice versa in case the imbalance minimizer is ‘Treatment B’. In case the imbalance minimizer is ‘Both’, chances are 50% for ‘Treatment A’ and 50% for ‘Treatment B’.

## Results

### Timeline of the platform development

In February 2020, the first confirmed SARS-CoV-2 infection was documented in Switzerland. On March 6th, the Swiss National Science Foundation opened a special call concerning SARS-CoV-2 related research topics. End of March 2020, our project for the set-up of a trial platform for the evaluation of preventive treatment options against SARS-CoV-2 in immunocompromised patients was submitted to this special call, with funding granted two months thereafter [23]. Without any efficient treatment or prevention option available at the time of funding approval, the study team started to draft a study protocol, and communication with interested SHCS and STCS study center representatives started. With the helpful input from various infectious disease specialists, transplant experts and literature research, the final research question and study endpoints took shape. Instead of investigating preventive treatment options against SARS-CoV-2, several vaccines were supposed to be explored in June 2020 when first preliminary phase I to II data became available with the antibody response and efficacy being the primary endpoints. In August 2020, implementation of the trial platform, including all technical aspects, started. At that time, no Covid-19 vaccine was licensed in Switzerland; hence the design was kept flexible to be adapted later on. The first Covid-19 vaccine was licensed in Switzerland on December 19th 2020 (BNT162b2 by Pfizer/BioNTech, brand name “Comirnaty” [24]), followed by the second licensed vaccine on January 12th 2021 (mRNA-1273 by Moderna, brand name “Spikewax” [25]). We decided to conduct a study based on these first two licensed vaccines and the trial platform was adapted accordingly. In parallel, the ethics approval was finalized and a first version of the master-protocol (without specifying the treatment arms at that submission stage) was submitted on December 15th 2020. We received feedback from the research ethics committee on January 12th 2021. In parallel, documents were sent to the legal departments of the participating local sites. On March 12th 2021, the protocol (master-protocol and first sub-protocol, defining the intervention arms) was re-submitted to the research ethics committee, and provisionally approved on April 1^st^ 2021. On April 15th, the last agreement was signed by the legal departments of the local sites, and the final documents re-submitted for ethics approval to the ethics. On April 19th, the final approval from the ethical committee was received. On the same day, the trial platform went to productive state and the first patient was randomized and vaccinated. Figure 1 summarizes the timeline of the implementation of this project.

### Feasibility and success of the platform

The decision to study the immune response of the first two licensed SARS-CoV-2 vaccines in Switzerland was taken in December 2020. The first patient was randomized on April 19th 2021, i.e., around five months later. On April 22nd, already 55 patients were randomized (> 40 patients) and on May 10th, 385 patients were randomized: Time to patient recruitment until 40 patients was hence four days, and time to patient recruitment until the anticipated study size of 380 patients was 22 days. The total number of patients recruited was 430, to account for potential losses to follow-up. With our trial platform, the consent rate could not be assessed, as patient information and consent documents were provided at the local study sites. Of the 430 patients, 412 could be included into the intention-to-treat dataset for the main analysis, i.e., comparing the two vaccines. The results of this pilot study, including detailed characteristics of the study population, were published on March 2nd 2022 [26]. In addition, data collected in the COVERALL pilot trial were merged back to data collected in the SHCS. With this, we could study determinants of antibody response to SARS-CoV-2 mRNA vaccines in people living with HIV, the corresponding manuscript was accepted on March 25th 2022 [27].

## Discussion

The main goal of this project was to develop a trial platform in order to react rapidly and efficiently to the fast moving research progress and public health guidelines during the evolving SARS-CoV-2 pandemic. Being challenged for more than one year with the inadvertent daily burdens this pandemic places on health care professionals, we emphasized on implementing the platform as user friendly as possible. In particular, we aimed to integrate the platform in already existing data infrastructures, making use of automatic data transfer and hence decreasing the administrative burden as much as possible. Already before the approval of the first SARS-CoV-2 vaccine in Switzerland and before the research question was well defined, we started setting up this trial platform to be able to study different aspects of the SARS-CoV-2 pandemic in immunocompromised patients. The main challenge was that the trial platform needed to be designed as flexible as possible: Nearly all study variables were undetermined at the time of planning and implementation, such as eligibility criteria, participation of transplant and HIV clinics, and clinical endpoints. The platform was hence designed in a modular and dynamic way, in order to be able to react fast to last-minute guideline and protocol changes before study launch.

To summarize the trial platform design, we created five major building blocks, each of them acting rather independently with the option for rapid adjustment: (1) A system to flag prioritized patients according to current guidelines; (2) Eligibility and inclusion criteria necessary for participation; (3) Randomization algorithm in order to balance groups with respect to the most important variables; (4) Electronic Case Report forms to collect study-specific data and (5) Cohort infrastructure to include all relevant information routinely collected during cohort follow-up visits.

We decided to perform randomization and new data collection using REDCap, a well-established and user friendly data collection tool. Design and adaptation of electronic case report forms in REDCap is very straightforward and can be done without knowledge of a programming language. REDCap has an in-build randomization and audit-track system necessary for randomized clinical trials. Moreover, REDCap offers great opportunities to extend it with further tools such as automatic data transfer and more sophisticated randomization algorithms, by using triggers and APIs [28]. For the monitoring of the study, REDCap offers in-build tools to create customized reports, enabling a convenient tool to observe the study progress and notice missing values and inconsistencies.

After the first and second SARS-CoV-2 vaccines were licensed in Switzerland, the final outline and scientific question of the COVERALL pilot trial for the trial platform was decided: The main goal was to study the efficacy of the two mRNA SARS-CoV-2 vaccines Comirnaty® and Spikewax®, the main outcome being antibody response. The main strength from the clinical perspective of this trial platform is the evaluation of the efficacy of the first two licensed SARS-CoV-2 vaccines in immunocompromised hosts in a randomized fashion, integrated within two well-established national cohort studies. Both the SHCS and STCS are prospective cohort studies with participants being enrolled for decades. Detailed clinical and epidemiological information of all participants is available, including knowledge about co-medication and laboratory parameters. In contrast to the other studies mentioned above, our study population is well-characterized with regular SHCS or STCS follow-up visits before and after the COVERALL trial. With this, it is possible to add further patient information later on when analyzing the main outcomes of our study, i.e., information about particular co-medication or comorbidities. Moreover, further blood samples are taken of all individuals in the course of regular follow-up visits, allowing to measure the SARS-CoV-2 antibody response at later time points. In addition, potential extensions of the COVERALL study, e.g., analyzing antibody responses after a third dose of vaccination, are easily doable without major technical obstacles, and are currently performed. The main strengths regarding technical innovation of this project certainly are that we were able to rapidly implement a flexible tool within two running systems: the dynamic set-up allowed for adjustment to the changing vaccine guidelines and allocation strategies. Within 6 weeks following the study launch, > 380 patients could be successfully included, allowing the study of immune response, efficacy and side effects of the two licensed SARS-CoV-2 vaccines in Switzerland in a randomized head-to-head comparison in immunocompromised patients.

During the implementation process, we faced some limitations of the trial platform. First, the risk category of the patients (low, medium, high risk) were determined at study launch and based on the latest laboratory results. For few patients newly registered to the SHCS or STCS, i.e., after COVERALL study launch, or with laboratory results coming in with a delay, the risk category needed to be defined and entered manually. Moreover, there was one patient registered in both cohort studies, i.e., an HIV-infected patient who had received a solid organ transplant. For this patient, we had to decide on one cohort and stratification system. In addition, we faced a problem with identifying patients in the STCS: In the trial platform, we worked with patient identifiers only, but the responsible study nurses at the different transplantation centers had not always access to these study identifiers, hence, re-identification using birth and transplantation dates was necessary for including the correct patients via the STCS patient identifier. A possible improvement of the trial platform would be to integrate a matching tool of patient information and patient identifiers to automatically identify patients.

In summary, in this project we rapidly developed a trial platform linking several tools available for medical data collection. We integrated the electronic system of the SHCS and the paper-based system of the STCS to collect new information using the well-established REDCap system. Taking the best of each system, we were able flag eligible patients, transfer patient information automatically, randomize and enroll the patients in an easy work flow, decreasing the administrative burden usually associated with a trial of this size.

## Data Availability

This manuscript describes the technical workflow of the trial platform. The source data, i.e., the implementation details in REDCap, the source scripts within the Django framework (Python scripts), and minimization algorithm (R Scripts), are embedded within the larger cohort infrastructure. In case of specific questions and reasonable requests, these source scripts are available from the corresponding author.
